# Body composition and obstructive sleep apnoea assessment in adult patients with Prader–Willi syndrome: a case control study

**DOI:** 10.1007/s40618-022-01831-5

**Published:** 2022-06-20

**Authors:** G. Pugliese, L. Barrea, A. Sanduzzi Zamparelli, G. de Alteriis, D. Laudisio, G. Muscogiuri, A. Canora, M. Bocchino, A. Colao, S. Savastano

**Affiliations:** 1 Dipartimento di Medicina Clinica e Chirurgia, Sezione di Endocrinologia, Università “Federico II” di Napoli, Via Sergio Pansini, 5, 80131 Naples, Italy; 2 Centro Italiano per la Cura e il Benessere del Paziente con Obesità (C.I.B.O), Dipartimento di Medicina Clinica e Chirurgia, Sezione di Endocrinologia, Università “Federico II” di Napoli, Via Sergio Pansini, 5, Naples, Italy; 3 Dipartimento di Scienze Umanistiche, Università Telematica Pegaso, Naples, Italy; 4 Dipartimento di Pneumologia, Ospedale di Alta Specializzazione ‘V. Monaldi’, Università “Federico II” di Napoli, Naples, Italy; 5 Cattedra Unesco “Educazione alla Salute e allo Sviluppo Sostenibile”, Università “Federico II” di Napoli, Naples, Italy

**Keywords:** Prader–Willi syndrome, Body composition, Sleep-breathing disorder, Obstructive sleep apnoea, Genetic obesity

## Abstract

**Introduction:**

In Prader–Willi syndrome (PWS) adult patients, sleep-breathing disorders, especially obstructive sleep apnoea syndrome (OSAS), are very common, whose missed or delayed diagnosis can contribute to further increase cardiovascular morbidity and mortality.

**Purpose:**

The aim of this cross-sectional study was to evaluate differences in sleep-breathing parameters obtained by overnight cardiorespiratory polygraphy in 13 adult PWS patients and 13 individuals with non-syndromic obesity as controls matched by age, sex, and BMI.

**Methods:**

In all subjects’ anthropometric parameters, body composition using bioimpedance analysis and overnight cardiorespiratory monitoring parameters were obtained.

**Results:**

Ten (76.9%) PWS patients were diagnosed with OSAS, most notably nine (69.2%) and one PWS (7.7%) with mild and severe OSAS, respectively. Compared with the control group, PWS patients had evidence of higher apnoea–hypopnea index (AHI) (*p* = 0.04) and oxyhaemoglobin desaturation index (ODI) (*p* = 0.009). However, no differences were found between the two groups regarding OSAS categories or diagnosis of nocturnal respiratory failure. In the PWS group, there were no significant correlations among AHI, ODI and hypoxemia index (T90) and anthropometric measurements, fat mass (FM), and FM percentage (%). Conversely, in the control group, the sleep-related respiratory indices evaluated correlated positively with BMI, waist circumference, FM and FM%.

**Conclusions:**

This study confirmed that AHI and ODI indices were worse in PWS than in age, sex and BMI-matched controls. The lack of their significant association with the anthropometric parameters and FM supported the existence of PWS-related mechanisms in OSAS pathophysiology that are independent of visceral obesity and FM.

## Introduction

Prader–Willi syndrome (PWS) is a rare genetic disorder caused by the lack of expression of genes on the paternally inherited chromosome 15q11.2-q13, with an estimated prevalence that ranges between 1/10,000 and 1/30,000 [1]. At birth, typical clinical manifestations include muscular hypotonia, feeding difficulties, retarded psychomotor development, and slowed growth with short stature. During childhood and adulthood, PWS patients generally develop pathologic hyperphagia responsible for severe obesity and early metabolic alterations, associated to mild-to-moderate intellectual and learning disabilities, behavioural and psychiatric symptoms, including obsessive–compulsive and oppositional behaviours [1–3].

Hypothalamic dysfunction is also a common finding in PWS that has been implicated in several clinical manifestations of the syndrome, including temperature instability, high pain threshold, sleep-breathing disorders, and endocrine abnormalities, such as growth hormone (GH) deficiency, central adrenal insufficiency, hypogonadism, and hypothyroidism [4]. These hormonal abnormalities contributed to the typical body composition pattern observed in PWS, which is characterised by higher fat mass (FM) than fat-free mass (FFM) compared with individuals with non-syndromic obesity matched for age and gender [5], reduced FFM and increased FM/FFM ratio when compared with both normal weight and individuals with obesity [6, 7]. However, in line with a recent paper by Grugni et al., the institution of GH replacement therapy during childhood could account for persistent improvement of body composition in adulthood [8].

The sleep-breathing disorder most frequently encountered in PWS patients is obstructive sleep apnoea syndrome (OSAS) [9]. OSAS is characterised by recurrent apnoeic events with increased upper airway resistance in the presence of respiratory effort, which leads to intermittent hypoxia and sleep fragmentation [10]. Polysomnography is the gold standard for diagnosis of OSAS [11].

However, polysomnography is a costly procedure with technical difficulties, which requires to be performed in a sleep laboratory. In addition, due to the variable grade of intellectual disability that characterise PWS, these patients not always are compliant or easily tolerate the complex polysomnographic recording devices.

Home overnight cardiorespiratory polygraphy is a reliable alternative to overnight polysomnography for the diagnosis of sleep-breathing disorders [12, 13]. It is of great clinical relevance to investigate the concomitant presence of OSAS in PWS, as OSAS represents an independent risk factor for hypertension, insulin resistance, and type 2 diabetes mellitus (T2DM) [9], which worsen the metabolic and cardiovascular risk in these patients.

In the general population, OSAS has a prevalence of 17%, which reaches 40–70% in individuals with obesity, and 58–86% in individuals with type T2DM [9].

Gender is a known potential moderator of OSAS prevalence in general population [14, 15]. In particular, among adults, the prevalence of OSAS was higher in men (13–33%) than in women (6–19%). However, to date, a gender difference in the prevalence of OSAS in PWS has not been demonstrated [16], as well as there are no significant gender differences in apnoea–hypopnea index (AHI) [17], or significant correlations between the sleep variables and gender in PWS patients [18]. According to recent studies, in children with PWS, the prevalence of OSAS is about 80–93% [16, 19], which is significantly higher than that observed in the general paediatric population (1–4%) [20]. In the adult population with PWS, the prevalence of OSAS reaches 95%, with a prevalence of 21% of severe OSAS, defined as presence of more than 30 apnoea or hypopneas events *per* hour [21].

In particular, obesity, altered ventilatory control, airway hypotonia, micrognathia, narrowing of the upper airway, and respiratory muscle weakness, are all common conditions in PWS that contribute to make these patients highly vulnerable to developing sleep-breathing disorders [22].

However, due to the psychomotor retardation that affects PWS patients, the symptoms of OSAS can be difficult to detect. Caregivers often report snoring, nocturnal breathing difficulties, and restless sleep, as well as marked daytime sleepiness [23]. However, if PWS patients are not carefully assisted by a multidisciplinary team, the diagnosis of OSAS can escape. The missed diagnosis of OSAS can have repercussions on both sleep and life quality, which contribute to further increase cardiovascular morbidity and mortality in PWS patients [24]. There is also evidence of sudden and unexplained death during sleep in patients with PWS, which may be secondary to respiratory pathology [25].

So far, studies analysing the differences between adult patients with PWS and non-syndromic obesity are limited*.* In particular, Yee et al. previously reported that in a sample cohort of adult and adolescent PWS patients compared with a control group matched on the basis of age, sex, and body mass index (BMI), PWS patients had more evidence of nocturnal hypoxemia, with lower saturations of oxyhaemoglobin and higher percentages of sleep time at less than 80% oxyhaemoglobin saturation [21].

The aim of this study was to investigate the differences in sleep-breathing parameters, in particular AHI, oxyhaemoglobin desaturation index (ODI) and hypoxemia index (T90), evaluated by overnight cardiorespiratory polygraphy, in a sample of adult PWS patients compared with age, sex and BMI-matched controls, and their possible association with body composition.

## Materials and methods

We carried out a cross-sectional observational study at the Department of Clinical Medicine and Surgery, Unit of Endocrinology, University Federico II, Naples (Italy). The study protocol, approved by the Ethical Committee of the University of Naples Federico II Medical School (n. 173/16), was carried according to the Code of Ethics of the World Medical Association (Declaration of Helsinki) for experiments involving humans and patients were widely informed before signing their consent. For the PWS group, subject consent was acquired through the primary caregiver or guardian.

Thirteen adult PWS patients with genetically confirmed diagnoses (via positive methylation testing) attending the Outpatient Clinic of the Unit of Endocrinology were consecutively recruited from October 2016 to January 2020 regardless of symptoms of OSAS. Ten patients received GH replacement therapy during childhood, while only three patients were diagnosed in adulthood and were not treated. GH therapy was withdrawn at least 10 years before being selected for this study.

Thirteen Caucasian age, sex and BMI-matched control subjects with obesity were consecutively recruited from the same geographical area around Naples metropolitan area, Italy, among subjects referred to the pneumology unit for snoring or other clinical features of OSAS, as daytime sleepiness, difficulty concentrating, and diurnal somnolence. None of them had previously diagnosed with OSAS or was treated with continuous positive airway pressure (CPAP). Subjects were excluded if they had any known lung disease or uncontrolled metabolic or cardiovascular diseases. Subjects were also excluded if they were on anti-obesity treatments, ketogenic diet, stimulant medications, medications that would affect sleep and breathing (e.g. opiates, benzodiazepines), or had significant alcohol intake. Since the study protocol included the assessment of body composition by bioimpedance analysis (BIA), we excluded subjects with pacemakers or defibrillators that could potentially interfere with BIA assessment, or patients suffering from chronic diseases that could interfere with fluid homeostasis, such as liver or chronic renal diseases, cancer, and acute or chronic inflammatory diseases.

All the anthropometric parameters were measured in the morning between 8:00 a.m. and 12:00 p.m. after an overnight fast, by an expert Nutritionist according to the International Society for the Advancement of Kinanthropometry of 2006 (ISAK 2006), with patients wearing light clothes and without shoes [26, 27]. The height was measured with a wall-mounted stadiometer (Seca 711; Seca, Hamburg, Germany), while weight was assessed by a calibrated balance beam scale (Seca 711; Seca, Hamburg, Germany). A no stretchable measuring tape was used to assess waist circumference (WC) by the same Nutritionist: tape was placed around the bare abdomen just above the hip bone and parallel to the floor and measurement was taken to the nearest centimetre at the midpoint between the bottom of the rib cage and above the top of the iliac crest during minimal respiration [28]. BMI was calculated using the following formula: weight (kg) divided by height squared (m^2^), and BMI was used to divide the subjects into obesity classes as established by World Health Organization’s criteria: grade I obesity (BMI: 30.0–34.9 kg/m^2^), grade II obesity (BMI: 35.0–39.9 kg/m^2^), and grade III obesity (BMI ≥ 40.0 kg/m^2^) [29].

On the same day, immediately after the measurement of the anthropometric parameters and, therefore, still in a fasting condition, body composition was assessed using a BIA phase-sensitive system by experienced observers (an 800-µA current at a frequency single-frequency of 50 kHz BIA 101 RJL, Akern Bioresearch, Florence, Italy). The exam was performed as suggested by the European Society of Parental and Enteral Nutrition (ESPEN) [30]. The electrodes were placed on the hand and the ipsilateral foot, according to Kushner [31]. The fat mass was obtained using the prediction equation for adult male PWS patients developed by Gray et al. [32] and for female PWS patients developed by Bedogni et al. [33], as already used in other works [34, 35]. The same operator and the same device obtained BIA determinations under strictly standardised conditions to avoid interobserver and inter-device variability. The BIA was routinely checked with resistors and capacitors of known values.

Overnight cardiorespiratory monitoring in ambient air was performed through the polygraph (Vital Night, Vitalaire, Rangendingen, Germany) with assessment of airflow through nasal cannula, respiratory effort by thoraco-abdominal bands and oxygen saturation and heart rate with pulse oximetry [36]. Cardiorespiratory monitoring measures apnoea events, that are defined as episodes of cessation of breathing of at least 10 s with or without oxygen desaturation, and hypopnea events characterised by a reduction of respiration of at least 10 s with a reduction of at least 30% of the nasal pressure signal. The number of apnoea and hypopnea events per hour of sleep was used to calculate the AHI. According to international guidelines, diagnosis of OSAS was made if there were an AHI ≥ 5/h. This index was also used to stratify the disease severity: mild with AHI ranging between 5 and 14/h, moderate with AHI between 15 and 29/h and severe with AHI ≥ 30/h [37]. ODI corresponds to the number of desaturations ≥ 4% per hour of recording and an ODI value > 10 raises the suspicion of OSAS. T90 was also evaluated, i.e. the percentage of time spent with oxygen saturation < 90%. If the T90 is > 30% of the recording time, nocturnal respiratory failure is diagnosed [36].

### Statistical analysis

Data of the study participants were expressed as means and standard deviations (SD) for normally distributed continuous variables and relative frequencies for categorical variables. The data normal distribution was evaluated by a Kolmogorov–Smirnov test, and the abnormal data were normalised by a logarithm. Skewed variables were back transformed for presentation in tables and figures.

Differences between adults PSW and control group were analysed by Student’s paired *t*-test. Chi square (χ^2^) test was used to determine the significance of differences in the variable frequency distribution across OSAS and ODI categories, and between nocturnal respiratory insufficiency (Y/N). The correlations between sleep-related respiratory indices and age, anthropometric parameters, and FM were assessed with Pearson *r* correlation coefficient. The level of significance was taken as *p* < 0.05. Data were analysed using the SPSS Software (PASW Version 21.0, SPSS Inc., Chicago, IL, USA) and MedCalc® package (Version 12.3.0 1993- 2012 MedCalc Software bvba—MedCalc Software, Mariakerke, Belgium.

## Results

The study population consisted of 26 participants: 13 adult PWS patients and 13 controls matched by age, sex, and BMI. Age, anthropometric characteristics, including WC as a surrogate measure of visceral adiposity, FM and FM percentage (FM%) of the study population are summarised in Table 1. There were no significant differences in anthropometric parameters or FM between PWS adults and controls.

**Table 1:** Age, anthropometric characteristics and fat mass in PWS adults and Control group

Parameters	PWS (*n* = 13)	Control group (*n* = 13)	*p* value
Males (*n*, %)	6 (46.1%)	6 (46.1%)	*χ*^2^ = 0.001 *p* = 1.0
Females (*n*, %)	7 (53.9%)	7 (53.9%)	*χ*^2^ = 0.001 *p* = 1.0
Age (years)	28 ± 6.8	29.9 ± 7.4	0.55
Weight (kg)	105.8 ± 36.5	122.9 ± 27.2	0.15
Height (m)	1.53 ± 0.11	1.65 ± 0.48	0.21
BMI (kg/m^2^)	44.2 ± 11.5	44.6 ± 9.1	0.89
WC (cm)	124.3 ± 29.5	114.8 ± 17.9	0.30
Fat mass (kg)	50.1 ± 25.9	63.3 ± 23.4	0.10
Fat mass (%)	45.4 ± 9.8	49.9 ± 9.1	0.06

*PWS* Prader–Willi syndrome, *BMI* Body mass index, *WC* waist circumference

Ten (76.9%) PWS patients were diagnosed with OSAS, nine (69.2%) and one PWS (7.7%) with mild and severe OSAS, respectively. OSAS prevalence did not differ significantly between PWS and control group (10/13 vs 6/13: *χ*^2^ 2.6, *p* = 0.11). Compared with the control group, PWS patients had evidence of higher AHI (*p* = 0.04) and ODI (*p* = 0.009). As reported in Table 2, no differences were found between the two groups regarding OSAS categories (mild, moderate, severe), diagnosis of nocturnal respiratory failure based on T90 and suspicion of OSAS based on ODI > 10.

**Table 2:** Overnight cardiorespiratory monitoring parameters and OSAS categories in PWS adults and Control group

Parameters	PWS (*n* = 13)	Control group (*n* = 13)	*p* value
Overnight cardiorespiratory monitoring parameters			
AHI (events/h)	9.2 ± 7.2	4.3 ± 2.6	***p* = 0.04**
ODI (events/h)	15.1 ± 8.9	7.3 ± 4.2	***p* = 0.009**
T90 (%)	10.6 ± 20.4	2.8 ± 1.8	0.195
OSAS (*n*, %)	10 (76.9%)	6 (46.1%)	*χ*^2^ = 2.6; 0.11
OSAS categories^a^			
No OSAS (*n*, %)	3 (23.1%)	7 (53.9%)	*χ*^*2*^ = 1.46; 0.22
Mild OSAS (*n*, %)	9 (69.2%)	6 (46.1%)	*χ*^2^ = 0.63; 0.42
Moderate OSAS (*n*, %)	0	0	
Severe OSAS (*n*, %)	1 (7.7%)	0	
ODI categories			
ODI < 10 (no suspicion of OSAS)	5 (38.5%)	7 (53.9%)	*χ*^2^ = 0.16; 0.69
ODI > 10 (suspicion of OSAS)	8 (61.5%)	6 (46.1%)	*χ*^2^ = 0.61; 0.43
Nocturnal respiratory failure			
T90 > 30%	1 (7.7%)	0	
T90 < 30%	12 (92.3%)	13 (100%)	

Significant *p* values are reported in bold

^a^According to international guidelines [37], a diagnosis of OSAS was made when AHI was ≥ 5/h, while the severity was classified as mild, moderate or severe when AHI was 5–14/h, 15–29/h, or ≥ 30/h, respectively

*PWS* Prader–Willi syndrome, *AHI* apnoea–hypopnea index, *ODI* oxyhaemoglobin desaturation index, *T90* hypoxemia index, *OSAS* obstructive sleep apnoea syndrome

### Correlation study

In the PWS group, there was no significant correlation in the sleep-related respiratory indices included in the study and anthropometric measurements, FM and FM% (Table 3). Conversely, in the control group, AHI correlated positively with BMI (*r* = 0.88; *p* = 0.001), WC (*r* = 0.73; *p* = 0.005), FM (*r* = 0.85; *p* = 0.001) and FM% (*r* = 0.76; *p* = 0.002); ODI correlated positively with BMI (*r* = 0.95; *p* = 0.001), WC (*r* = 0.84; *p* = 0.001), FM (*r* = 0.93; *p* = 0.001) and FM% (*r* = 0.82: *p* = 0.001); T90 correlated positively with BMI (*r* = 0.94; *p* = 0.001), WC (*r* = 0.96; *p* = 0.001), FM (*r* = 0.95; *p* = 0.001) and FM% (*r* = 0.86; *p* = 0.001).

**Table 3:** Correlations between sleep-related respiratory indices and anthropometric parameters and fat mass in PWS and controls

Parameters	AHI (events/h)		ODI (events/h)		T90 (%)	
	*r*	*p*	*r*	*p*	*r*	*p*
PWS group						
Age	0.09	0.75	0.43	0.14	0.44	0.12
BMI	0.04	0.88	0.54	0.06	0.14	0.63
WC	0.09	0.76	0.52	0.06	0.29	0.32
FM(kg)	0.05	0.85	0.51	0.07	0.29	0.32
FM (%)	− 0.11	0.72	0.51	0.07	0.29	0.32
Control group						
Age	− 0.26	0.37	− 0.09	0.75	− 0.11	0.71
BMI	0.88	**0.001**	0.9	**0.001**	0.94	**0.001**
WC	0.73	**0.005**	0.84	**0.001**	0.96	**0.001**
FM (kg)	0.80	**0.001**	0.93	**0.001**	0.95	**0.001**
FM (%)	0.76	**0.002**	0.82	**0.001**	0.86	**0.001**

Significant *p* values are reported in bold

* PWS* Prader–Willi syndrome, *AHI* apnoea–hypopnea index, *ODI* oxyhaemoglobin desaturation index, *BMI* Body mass index, *WC* waist circumference, *FM* fat mass

## Discussion

In line with the current literature [21], in our study, we found a high prevalence of OSAS in adult PWS patients, although without significant differences compared with a well-matched obese control population. As novel finding, we showed that there were significant differences in sleep-related respiratory indices obtained by the home overnight cardiorespiratory polygraphy between PWS patients and controls. In particular, PWS patients had a higher AHI and more frequent episodes of oxygen nocturnal desaturation compared with controls. Of interest, while in controls, the sleep-related respiratory indices were positively associated with anthropometric parameters, including WC as a surrogate measure of visceral adiposity, and fat mass, these correlations were not evident in PWS patients.

Our findings are in line with those of Marzullo et al., who demonstrated a significantly higher number of apnoea–hypopnea episodes and greater nocturnal desaturation in PWS patients than controls with non-syndromic obesity [38]. Moreover, our data confirm that the prevalence of OSAS is high among adult PWS patients, albeit not different compared to controls. However, while PWS patients performed the nocturnal respiratory monitoring without specific symptom of OSAS as part of the screening protocol provided by the PWS management guidelines, controls were recruited by the pneumology unit for suspected symptoms of OSAS.

In addition, we also observed a major impairment of sleep-related respiratory indices in PWS patients compared with controls. The major impairment of sleep-related respiratory indices in PWS patients let us to speculate that, in addition to the known risk factors for OSAS shared by PWS and non-syndromic obesity, such as excess weight, visceral obesity, and pulmonary restriction, there are PWS-related factors not normally present in subjects with non-syndromic obesity, such typical body composition pattern [5], respiratory muscle weakness, chest wall deformities, severe scoliosis, and craniofacial abnormalities, which could contribute to worsen the respiratory function in PWS patients and that therefore deserve to be investigated with ad hoc studies [22].

The independent relationship of the major impairment of sleep-related respiratory indices and obesity in PWS patients is in line with the similar independent relationship of ventilatory control responses to hypoxemia and hypercapnia and obesity in these patients. In particular, when breathing air with a reduced percentage of oxygen compared to the ambient air, 35% of PWS patients did not exhibit a hypoxic ventilatory response, while the remaining patients had a significantly blunted response compared to healthy controls [39]. Similarly, PWS patients had a blunted hypercapnic ventilatory response compared to non-obese PWS patients and BMI-matched obese controls, as further impediment in maintaining normoxia and normocapnia in an efficient manner [39]. Therefore, the ventilatory response to both hypoxia and hypercapnia appears to be reduced in PWS patients regardless of BMI, and the response to hypercapnia appears to be altered even during sleep [39], thus suggesting that abnormal arousal response or different thresholds to hypoxia and hypercapnia may be another contributing factor to OSAS in PWS.

In this complex scenario, it is tempting to speculate that the higher nocturnal desaturation in our sample of adult PWS patients compared with BMI-matched controls could unravel abnormalities in the brain centres that regulate sleep and breathing in PWS. In particular, genes identified at the neuronal level in animal models, including Necdin and Necdin-related MAGE, and involved in the control of respiration, are imprinted genes, whose paternal expression is absent in PWS [40]. In fact, it has been observed that Necdin deficiency alters the serotonergic metabolism, the morphology of serotonin vesicles in medullary serotonergic neurons, and that Necdin deficiency in mice induces central respiratory deficits reminiscent of PWS (irregular rhythm, frequent apnoeas, and blunted respiratory regulations) [40]. This experimental evidence suggested that the lack of Necdin could affect maturation and function of the respiratory network in PWS.

Furthermore, a dysfunction of the orexin system seems to be present both in animal models [41] and in PWS patients [42], which might be involved in the sleep-breathing disorders in PWS. Orexin is a neuropeptide exclusively produced in the perifornical area and the lateral and posterior hypothalamic area of the brain. Orexin neurons project throughout the central nervous system to nuclei involved in the control of sleep–wakefulness, feeding, neuroendocrine homeostasis, and autonomic regulation [43]. Recent evidence showed moderately decreased cerebrospinal fluid orexin levels in PWS patients [44], although the number of orexin neurons in the hypothalamus was not reduced in patients with PWS [45]. Therefore, the orexin deficiency in PWS appears to be more related to the disruption in the connecting pathway between the hypothalamus, the cerebral cortex, and the brainstem [45], rather than to the reduction in the number of orexin neurons. Interestingly, experimental studies on mouse models with orexin deficiency [46, 47] reported both a modulatory effect of the hypothalamic orexin system on respiratory activity [48], and an increase in spontaneous sleep apnoeas, especially in REM sleep [49], thus suggesting that the orexin dysfunction found in PWS may partly contribute to the pathophysiology of sleep-breathing disorders, commonly present in this syndrome.

Several studies have shown a relationship between increased BMI and both severity of OSAS and greater AHI in general population [16, 50]. As above mentioned, and in agreement with previous observations [19, 51], our findings confirmed that in our group of PWS patients there were no correlations between AHI and BMI or other anthropometric parameters included in the study. However, considering that BMI is a rather inaccurate measure of adiposity that does not allow to discriminate between fat mass and free fat mass, we included the assessment of fat mass obtained using BIA, and demonstrated that in PWS patients AHI and the other saturation parameters did not correlate with fat mass. Indeed, we found that there was a trend in the differences in FM% between PWS patients and controls, with a lower FM% in PWS patients. In line with the current literature [8], this finding lets us to hypothesise that the institution of the GH therapy during childhood in the vast majority of PWS patients included in our study group could account for the persistent improvement of body composition in adulthood in comparison with age and BMI-matched controls.

Regarding mortality in PWS patients, excluding the condition of sudden death with unknown cause, respiratory failure was the most common cause accounting for 31% of all deaths, followed by cardiac disease/failure (16%) [52]. These data suggest a possible reciprocal pathophysiological influence between the two systems in PWS patients, thus supporting the need for a complete cardiorespiratory evaluation. In particular, OSAS could be a potential contributor to cardiovascular alterations in PWS. In the general population, OSAS is an independent risk factor for arrhythmias, sympathetic hyperactivity, hypertension, diastolic and systolic dysfunction, pulmonary congestion, and cardiac hypertrophy [53]. In PWS patients sleep apnoea-related hypoxia is acknowledged as a cause of *cor pulmonale* [25, 54]. In addition, PWS patients have a lower left ventricle mass compared to subjects with non-syndromic obesity, and this aspect seems to be only partially dependent on GH deficiency, responsible for a hypotrophic hypokinetic syndrome. In particular, both Insulin-like growth factor-I levels and nocturnal oxygen desaturation were main significant predictors of left ventricle mass and heart rate in PWS patients [38]. To further underline the bidirectional link between OSAS and cardiovascular disease in PWS, it is reported in the literature that the use of adaptive servoventilation can improve the prognosis in case of heart failure in PWS secondary to sleep-disordered breathing [55]. These data supported to include the diagnosis and therapy of OSAS in the prevention of cardiovascular risk in PWS.

In non-syndromic obesity, there is also consolidated evidence regarding the effect of CPAP not only on the improvement of sleep-breathing parameters, but also of metabolic and cardiovascular risk factors [9]. CPAP treatment is also recommended in PWS patients with OSAS [56]. In a small cohort of PWS patients and in some case reports, CPAP was effective in improving OSAS-related symptoms, particularly excessive daytime sleepiness [51, 57–59]. However, the cardiometabolic effects of CPAP treatment in PWS patients have not yet been studied.

There are some limitations to this study. First, the cross-sectional nature of this study did not allow any statements on the causal relationships between OSAS and adiposity in PWS. Second, the sample size was relatively small, and this is because PWS is a rare disease. Nevertheless, we have calculated the sample size using 95% power to enrol the appropriate number of patients for each group, matching also controls for gender, age, and BMI. Third, we performed the overnight cardiorespiratory monitoring and did not the polysomnographic recording, so we are not able to determine accurately sleep stage and hypopneas linked to arousal. As a final limitation of the study, BIA is not the most accurate method for assessing body composition, although it is a commonly used and validated method in the clinical practice, with a high agreement with Dual X-ray Absorptiometry, also among patients with severe obesity [60]. However, the main strength of this study was the use of a control group age, sex and BMI-matched that allowed us to assess whether PWS per se was associated with worse sleep-related respiratory indices regardless of the obesity condition.

## Conclusion

In conclusion, we reported there were significant differences in sleep-related respiratory indices obtained by the home overnight cardiorespiratory polygraphy between PWS patients and a well-matched obese control population. Of interest, the alterations in sleep-related respiratory indices were not associated with anthropometric parameters, including WC, as a surrogate measure of visceral adiposity, and fat mass, thus supporting the existence of PWS-related OSAS pathophysiological mechanisms independent of visceral obesity and fat mass. Further studies with polysomnographic recording are mandatory to confirm this hypothesis.

## Data Availability

The datasets generated during and/or analysed during the current study are available from the corresponding author on reasonable request.

## Abbreviations

PWS: Prader–Willi syndrome
GH: Growth hormone
FM: Fat Mass
FFM: Fat-Free Mass
OSAS: Obstructive sleep apnoea syndrome
T2DM: Type 2 diabetes mellitus
BMI: Body mass index
BIA: Bioimpedance analysis
WC: Waist circumference
AHI: Apnoea–hypopnea index
ODI: Oxyhaemoglobin desaturation index
T90: Hypoxemia index
CPAP: Continuous positive airway pressure
IL-1β: Interleukin-1β
TNFα: Tumour necrosis factor-α
